# A Novel Bone Morphogenetic Protein 2 Mutant Mouse, nBmp2NLS^tm^, Displays Impaired Intracellular Ca^2+^ Handling in Skeletal Muscle

**DOI:** 10.1155/2013/125492

**Published:** 2013-11-28

**Authors:** Laura C. Bridgewater, Jaime L. Mayo, Bradley G. Evanson, Megan E. Whitt, Spencer A. Dean, Joshua D. Yates, Devin N. Holden, Alina D. Schmidt, Christopher L. Fox, Saroj Dhunghel, Kevin S. Steed, Michael M. Adam, Caitlin A. Nichols, Sampath K. Loganathan, Jeffery R. Barrow, Chad R. Hancock

**Affiliations:** ^1^Department of Microbiology and Molecular Biology, Brigham Young University, 775-A WIDB, Provo, UT 84602, USA; ^2^Department of Nutrition, Dietetics, and Food Science, Brigham Young University, Provo, UT 84602, USA; ^3^Department of Physiology and Developmental Biology, Brigham Young University, Provo, UT 84602, USA

## Abstract

We recently reported a novel form of BMP2, designated nBMP2, which is translated from an alternative downstream start codon and is localized to the nucleus rather than secreted from the cell. To examine the function of nBMP2 in the nucleus, we engineered a gene-targeted mutant mouse model (nBmp2NLS^tm^) in which nBMP2 cannot be translocated to the nucleus. Immunohistochemistry demonstrated the presence of nBMP2 staining in the myonuclei of wild type but not mutant skeletal muscle. The nBmp2NLS^tm^ mouse exhibits altered function of skeletal muscle as demonstrated by a significant increase in the time required for relaxation following a stimulated twitch contraction. Force frequency analysis showed elevated force production in mutant muscles compared to controls from 10 to 60 Hz stimulation frequency, consistent with the mutant muscle's reduced ability to relax between rapidly stimulated contractions. Muscle relaxation after contraction is mediated by the active transport of Ca^2+^ from the cytoplasm to the sarcoplasmic reticulum by sarco/endoplasmic reticulum Ca^2+^ ATPase (SERCA), and enzyme activity assays revealed that SERCA activity in skeletal muscle from nBmp2NLS^tm^ mice was reduced to approximately 80% of wild type. These results suggest that nBMP2 plays a role in the establishment or maintenance of intracellular Ca^2+^ transport pathways in skeletal muscle.

## 1. Introduction

Bone morphogenetic proteins (BMPs) are members of the transforming growth factor *β* (TGF-*β*) super family, and the first members of the family were identified by their ability to induce ectopic bone formation in animals [1, 2]. BMPs have since been shown to participate in multiple developmental pathways, including axis formation, limb patterning, heart development, neural crest cell migration, neurogenesis, apoptosis, and others [3–12].

The BMP family of proteins is the largest subfamily in the TGF-*β* superfamily, containing over twenty members including the growth and differentiation factors (GDFs) [5, 13]. BMP proteins are synthesized as preproproteins, which are directed by N-terminal signal peptides to the rough endoplasmic reticulum (ER) for translation, processing, and secretion from the cell. While in the secretory pathway, BMP proproteins homodimerize by disulfide bonding and are cleaved by furin-type proprotein convertase enzymes to produce the mature secreted BMP growth factors [14, 15]. Secreted BMPs bind to cell surface receptors and trigger cellular responses through the SMAD and the mitogen-activated protein kinase (MAPK) pathways [16–19].

Secreted BMP growth factors have been widely studied since their discovery over two decades ago. Recently, however, we found that some BMP family members can be translated in a novel alternative form that is translocated to the nucleus rather than being secreted from the cell [20]. BMP2, BMP4, and GDF5 all have nuclear variants, which we named nBMP2, nBMP4, and nGDF5, respectively. Nuclear localization is mediated in each case by a bipartite nuclear localization signal (NLS) that overlaps the furin proprotein convertase cleavage site. Cleavage at this site would destroy the NLS, but because these proteins are translated from an alternative downstream start codon and thus lack the N-terminal signal peptide, they are translated in the cytoplasm rather than the ER and thus avoid contact with the proprotein convertases in the Golgi apparatus. The intact NLS directs nuclear translocation [20].

The conservation of nuclear variants among three distinct BMP family members suggested a physiologically important role for these novel nuclear proteins [20]. To evaluate the *in vivo* requirement for nBMP2, we generated  nBmp2NLS^tm^  targeted mutant mice, which produce nBMP2 that cannot translocate to the nucleus. The conventional BMP2 growth factor, however, is still secreted and functions normally. Here we report that the  nBmp2NLS^tm^  mouse exhibits impaired skeletal muscle relaxation rates, suggesting a defect in the intracellular transport of Ca^2+^.

## 2. Materials and Methods

### 2.1. Cell Culture and Transfections

Rat chondrosarcoma (RCS) cells were maintained in Dulbecco's Modified Eagle's Medium (DMEM) supplemented with penicillin (50 u/mL), streptomycin (50 *μ*g/mL), L-glutamine (2 mM), and 10% fetal bovine serum at 37°C under 5% CO_2_. Cells were passaged every 3-4 days.

To determine the effect of the **RKR** to **AAA** NLS mutation on the nuclear localization of nBMP2, wtBmp2/GFP, or RKRmBmp2/GFP fusion plasmids (both containing the GFP tag at the C-terminus of BMP2) were transfected into RCS cells on Lab-Tek II Chamber Slides (ISC Bioexpress) using the *Trans*IT-Jurkat Transfection Reagent (Mirus, Madison, WI) according to the manufacturer's instructions. 48 hrs after transfection, cells were fixed using 4% paraformaldehyde, nuclei were stained with a 1 : 1000 dilution of TOPRO-3 iodide (Invitrogen Corporation, Carlsbad, CA), and slides were mounted in Fluoromount-G (Southern Biotech, Birmingham, AL) and coverslipped. Cells were imaged and nuclear localization was quantified using an Olympus IX81 laser confocal microscope as previously described [20].

To assess BMP2 secretion, RCS cells were seeded in 25 cm^2^ culture flasks and transfected with HA-tagged expression plasmids wtBmp2/HA or RKRmBmp2/HA (both containing the HA tag at the C-terminus of BMP2) as described above. After 48 hrs, culture medium was collected and HA-tagged proteins that had been secreted into the culture medium were precipitated using EZview Red Anti-HA Affinity Gel (Sigma, Saint Louis, MO) according to the manufacturer's protocol to concentrate the proteins. Precipitated proteins were separated by SDS-PAGE and analyzed by immunoblot using an anti-HA primary antibody.

### 2.2. Research Animals

Experimental procedures were approved by the Institutional Animal Care and Use Committee (IACUC) of Brigham Young University. Mice were kept in a temperature-controlled (21-22°C) room with a 12 : 12 hr light-dark cycle and were fed standard rodent chow and water ad libitum.

### 2.3. Construction of the nBmp2NLS^tm^ Targeting Vector

The targeting vector was constructed using “recombineering,” as described in [21]. Briefly, a genomic clone containing the BMP2 gene was retrieved from the BAC RP23-384M14 (The BACPAC Resource Center, Children's Hospital Oakland Research Institute, Oakland, CA). The BMP2 bipartite NLS was mutated by first replacing the target sequence with the *gal*K gene and then replacing the *gal*K gene with the desired mutation to alter the protein sequence of the NLS (KREKRQAKHKQ**RKR**LK was changed to KREKRQAKHKQ**AAA**LK) as described in [22]. Specifically, the nucleotide sequence 5′-CGG AAG CGC (coding for **RKR**) was replaced with 5′-GCG GCG GCC (coding for **AAA**).

The mutant nBMP2 gene was then retrieved from the BAC into pBluescript modified to contain a thymidine kinase gene (*MC1TK*). A male germline, self-excising neo cassette (tACN) was also inserted into the targeting construct between the BMP2 homology arms [23].

### 2.4. Generation of the nBmp2NLS^tm^ Mouse

The  nBmp2NLS^tm^  targeting vector was linearized and electroporated into 129/Bl6 G4 ES cells (generously provided by Andras Nagy of the Samuel Lunenfeld Research Institute in Toronto, Canada), and targeted cell lines were subjected to positive/negative selection as described by Mansour et al. [24, 25]. Cells were analyzed by Southern blot to determine the presence of the targeted nBMP2-mut allele. Two targeted cell lines were then microinjected into Bl6 host blastocysts, and both gave rise to chimeras. Chimeric males were intercrossed with Bl6 females to generate  nBmp2NLS^tm+/−^  mice. Heterozygous mice were intercrossed to produce the male − /−  (mutant) and + /+  (control) mice utilized in this study. Successful self-excision of the neo cassette (tACN) was verified by PCR using primers that annealed to the BMP2 gene regions bracketing the neo cassette (forward primer: CCTGCAGCAAGAACAAAGCAGG; reverse primer: CCCCAACCTTGTCATCATTCACC.) PCR product size from wild type was 525 bp; after insertion of the targeting vector and self-excision of the neo cassette it was 607 bp.

### 2.5. Genotyping

To distinguish the genotypes of progeny from heterozygous intercrosses, we performed two PCR reactions on DNA samples from each offspring. One reaction employed a primer set that detected the presence of the wild type allele, whereas the second identified the  nBmp2NLS^tm^  allele. Primers sets for both reactions used the same forward primer, but the reverse primer for the wild type reaction bound only the wild type DNA sequence and the reverse primer for the mutant reaction bound only the mutant DNA sequence. DNA from wild type mice yielded the 201 bp product only in the wild type reaction, DNA from homozygous mutants yielded the same size product only in the mutant reaction, and DNA from heterozygotes yielded the product in both reactions. Sequences for the primers were as follows: forward primer: GGCCCATTTAGAGGAGAACC; wild type reverse primer: TTGCAGCTGGACTTGAGGCGCTTCCG; mutant reverse primer: TTGCAGCTGGACTTGAGGGCCGCCGC. The 9 bp sequence that was altered to convert **RKR** to **AAA** is underlined. Reaction conditions are 94°C 5 min, 94°C 20 sec, 61.5°C 30 sec, and 68°C 40 sec, repeated 30x.

### 2.6. Skeletal Morphometric Analysis

Two wild type and three homozygous mutant male mice, 9-10 weeks old, were euthanized by CO_2_ inhalation and eviscerated, soft tissues were removed in 1% KOH, and skeletons were stained in a 0.004% Alizarin red in 1% KOH solution and then cleared in a graded glycerin series (20%, 50%, 80%, and 100%). The length and the proximal, distal, and midshaft widths at both the widest and narrowest diameters of the femur, tibia, humerus, and radius, both left and right limbs, were measured using digital calipers. Measurements were also taken of the scapula, pelvis, sternum, rib cage, skull, and vertebral column.

### 2.7. Grip/Strength Test

Male mice, five homozygous wild types and five homozygous mutants, were analyzed using the grip/strength test at  20.6 ± 0.3  weeks (wild type) and 20.7 ± 0.3 weeks (mutant) of age for the first measurement, and measurements were repeated once a week for six weeks. Each mouse was placed on a wire mesh cage lid, which was then inverted over the open cage filled with bedding, and mouse was held suspended approximately 25 cm above the bedding [26]. Holding time was recorded up to a maximum of 3 min.

### 2.8. Western Analysis of Muscle BMP2

To compare production of conventional BMP2 in wild type versus  nBmp2NLS^tm^  mouse skeletal muscle, cytoplasmic extracts were prepared from gastrocnemius and quadriceps muscle by homogenizing tissue in ice-cold buffer (0.25 M sucrose, 10 mM NaCl, 3 mM MgCl_2_, 1 mM DTT, 1 mM PMSF, and a protease inhibitor cocktail), centrifuging for 5 min at 500 ×g, and collecting supernatant. Protein concentration was quantified by Bradford assay before western blot analysis using BMP2 primary antibody N-14 from Santa Cruz (product #sc-6895). Autoradiograms were scanned and bands quantified using AlphaEase software.

### 2.9. Immunohistochemistry

Gastrocnemius muscle from 6-month-old male wild type and mutant mice was isolated, embedded in paraffin, and cross-sectioned at 6 *μ* thickness. After deparaffinization and rehydration, sections were stained with a biotin-tagged primary antibody NBP1-19751B (Novus Biologicals). Staining was visualized using the LSAB+ System-HRP kit from Dako, with a hematoxylin counterstain. Sections were viewed and imaged using a Zeiss Imager A.1 microscope with an AxioCam HRC camera and AxioVision 4.7 imaging software.

### 2.10. *In Situ* Muscle Preparation

Male mice were used in the muscle stimulation experiments at  50.9 ± 0.4  weeks (control) and  43.9 ± 7.5  weeks (mutant) of age. Muscle preparation was similar to that described previously [27–29]. Briefly, mice were anesthetized with 70 mg/kg intraperitoneal injection of sodium pentobarbital. The hamstrings were cut away from the gastrocnemius, plantaris, and soleus (GPS) muscle complex and the femur was secured on both the medial and lateral sides of the knee by two 16-gauge pins to prevent movement. The foot was also clamped to the platform to eliminate movement of the lower leg. The Achilles tendon was then secured to a Grass Force-Displacement Transducer FT03 level arm with a calibrated tension of 700 grams. The sciatic nerve was exposed, tied off, and cut. An electrode was placed directly on the sciatic nerve to achieve stimulation. Animals were supported with 100% oxygen directly to the nose throughout the procedure.

### 2.11. *In Situ* Isometric Contractions

The GPS muscle complex was stimulated via electrical stimulation (2-3 V stimulation, 0.05 ms square wave, at a frequency of 150 Hz, with the use of a Grass S88X Stimulator). Both a force frequency analysis and a 2 Hz twitch contraction protocol were used. A force frequency curve was developed by stimulating the muscle for ten pulses at varying frequencies (10, 20, 40, 60, 80, 100, 120, and 140 pulses/sec). The percent of maximal tetanic force production was determined over all frequencies tested. In order to examine the capacity for sustained muscle contractions, twitch contractions at the rate of 2 contractions/sec for 10 minutes were elicited.

### 2.12. Contractile Function

The GPS complex data was analyzed using LabScribe2 by iWorx, which captured data at 1000 Hz. The muscle was stretched to achieve maximal force as previously described [28]. Several tetanic contractions were elicited to stretch the muscle to the length that created maximal force. Peak twitch force was evaluated, as well as one-half relaxation times, every 60 pulses for 10 minutes. Relaxation was determined by measuring the time required for the GPS muscle to relax to 50% of the peak force for individual contractions.

### 2.13. SERCA Enzyme Activity Assay

#### 2.13.1. SR Membrane Purification

Sarcoplasmic reticulum (SR) membranes were prepared and purified as described by Kosk-Kosicka, with minor adaptations for small muscle samples [30]. All steps were performed at 4°C unless otherwise specified. Skeletal muscle was removed from limbs and washed in 0.1 mM EDTA pH 7.0 and then homogenized in Solution 1 (10 mM MOPS, 10% sucrose, 0.1 mM EDTA pH 7.0) and pH was adjusted to between 6.5 and 7.0 using 10% NaOH. Samples were centrifuged at 15,000 ×g for 20 min. Supernatant was collected and filtered through one layer of gauze then centrifuged at 40,000 ×g for 90 min. The pellet was resuspended in Solution 2 (10 mM MOPS, 0.6 M KCl, pH 7.0) and allowed to incubate at room temperature for 40 min. Again, the preparation was centrifuged at 15,000 ×g for 20 min, and the supernatant was collected and centrifuged at 40,000 ×g for 90 min. The resulting pellet was resuspended in 1 mL of Solution 3 (10 mM MOPS, 30% sucrose, pH 7.0). Protein concentrations were determined using a standard Bradford protein assay, and preparations were quick-frozen in liquid nitrogen for short-term storage.

#### 2.13.2. Measurement of SERCA Activity

SERCA activity was measured at both 15 and 30 min reaction time points for every sample, and both Ca^2+^ dependent (assay buffer: 50 mM Tris-maleate, pH 7.4; 8 mM MgCl_2_; 120 mM KCl; 1 mM EGTA; 10 *μ*M ionophore A23187; 1.008 mM CaCl_2_ to yield 17.5 *μ*M free Ca^2+^) and Ca^2+^ independent (assay buffer: 50 mM Tris-maleate, pH 7.4; 8 mM MgCl_2_, 120 mM KCl; 1 mM EGTA; 10 *μ*M ionophore A23187) reactions were performed as described by Kosk-Kosicka [30]. Briefly, 0.2 *μ*g of SR membrane preparation was added to each tube and enough water was added to give a final volume of 10 *μ*L. 85 *μ*L of the appropriate membrane assay buffer was then added to each tube. Reactions were started 15 sec after the buffer was added by adding 5 *μ*L 60 mM ATP, and reactions were capped, vortexed, and placed in a 37°C water bath. Reactions were stopped precisely 15 or 30 min after ATP was added by adding 300 *μ*L Lin Morales Reagent (see [30] for reagent composition) and vortexing. Results were measured as absorbance at a wavelength of 350 nm, precisely 30 sec after the addition of Lin Morales Reagent. The Ca^2+^ independent and dependent reactions were performed so that the base ATPase activity (Ca^2+^ independent) could be subtracted from Ca^2+^ dependent ATPase activity. Results of mutant were normalized to wild type SERCA activity.

### 2.14. Data Analysis

Analyses of data from grip/strength tests, morphometric measurements, *in situ* isometric contractions, contractile function, and SERCA enzyme activity experiments were performed using two-tailed, unpaired Student's  *t*-tests assuming unequal variance. Significance was set at  *P* < 0.05.

## 3. Results

### 3.1. nBmp2NLS^tm^ Mouse Construction

Mouse embryos lacking all BMP2 activity have been previously described [8]. These embryos died at approximately 7 days of development due to defects in the formation of the chorion and amnion [8]. In order to examine the effects of nBMP2 inactivation separately from the complete BMP2 knockout, it was necessary to devise a mutation scheme that would leave the conventional secreted form of BMP2 intact while preventing the function of nBMP2. We previously demonstrated in tissue culture that mutating the alternative start codon from which nBMP2 translation initiates only results in a 50% reduction of nBMP2 relative to controls, suggesting that other alternative start sites can be used to generate forms of BMP2 that localize to the nucleus if the primary alternative start site is mutated [20]. Mutation of the alternative start site, therefore, was unlikely to abolish nBMP2 in a mouse model.

**Figure 1:**
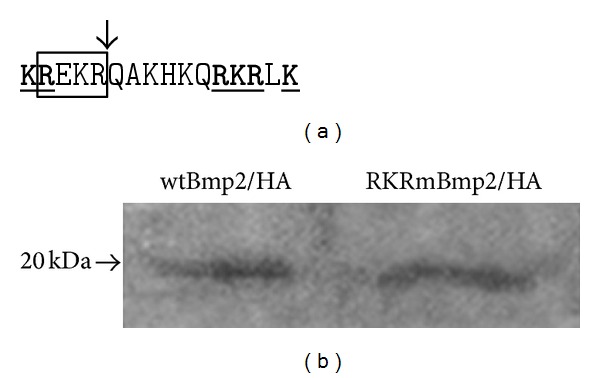
The **RKR** to **AAA** mutation in the bipartite NLS does not inhibit secretion of the conventional BMP2 growth factor. (a) Detail of the nBMP2 NLS sequence. Critical amino acids in the bipartite NLS are bold and underlined, the furin recognition site is boxed, and the site of cleavage to release the mature secreted growth factor from the propeptide is marked by an arrow. (b) Culture media from cells transfected with either wild type (wtBmp2/HA) or mutant (RKRmBmp2/HA) BMP2 expression plasmids was collected and analyzed by western blot. The HA-tagged growth factors were secreted equally.

Instead, we made specific alterations to the portion of the nBMP2 gene that encodes the bipartite NLS, whose alterations were predicted to block nuclear translocation of nBMP2 yet still allow synthesis and secretion of conventional BMP2. A consensus bipartite NLS is characterized by the following pattern: two basic residues, approximately 10 spacer residues, and another basic region consisting of 4 basic residues out of five. The sequence of the nBMP2 NLS is shown in Figure 1(a). The upstream **KR** portion of this NLS sequence overlaps the R-X-(K/R)-R furin recognition sequence where the BMP2 proprotein is cleaved to release the mature growth factor, and mutation of the **KR** would thus disrupt production of the secreted growth factor (Figure 1(a)). The downstream basic **RKRLK** portion of the BMP2 NLS, however, does not affect propeptide cleavage. To determine whether mutation of this portion of the NLS was sufficient to prevent nuclear localization, we constructed a mutant BMP2/GFP expression plasmid (called RKRmBmp2/GFP) in which **RKR** was replaced with **AAA** and transfected it into cultured cells. This mutation eliminated nuclear localization of the GFP-tagged BMP2 in cultured RCS cells just like the previously described **KR RKR** to **AA AAA** mutation did [20]. To determine whether BMP2 growth factor containing this mutation could still be secreted, an HA-tagged RKRmBmp2 expression vector (RKRmBmp2/HA) was transfected into cultured RCS cells and culture medium was collected 48 hrs later. HA-tagged proteins were immunoprecipitated from the medium and visualized by immunoblotting. The medium from cells transfected with RKRmBmp2/HA contained as much HA-tagged BMP2 growth factor as medium from cells transfected with the wild type wtBmp2/HA plasmid, indicating that the **RKR** to **AAA** did not disrupt synthesis or secretion of the conventional BMP2 growth factor (Figure 1(b)).

**Figure 2:**
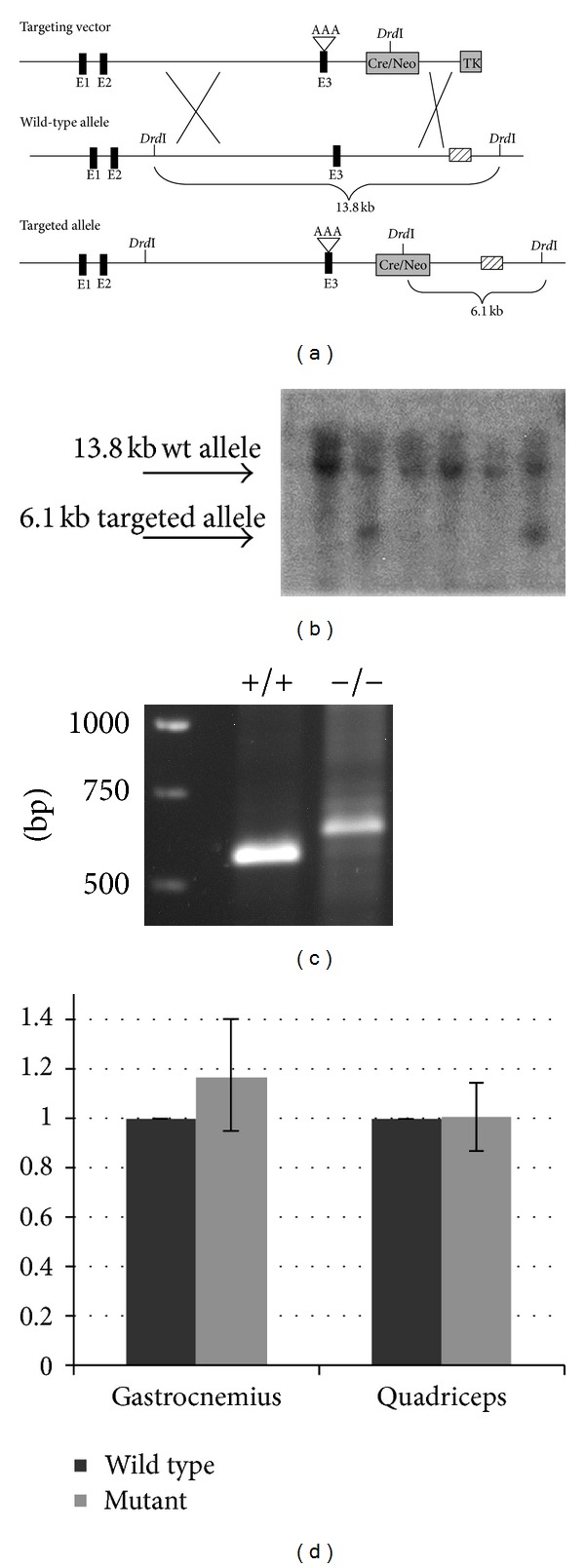
Integration of the  nBmp2NLS^tm^  targeting vector. (a) Schematic of the nBmp2NLS^tm^ targeting vector recombining into genomic DNA. The *BMP2* exons are numbered and represented as black boxes, while the *neo* cassette and thymidine kinase (TK) gene are represented as grey boxes. Insertion of the *neo* cassette introduces a *Drd*I restriction site as indicated. The mutation of the bipartite NLS (**KR**EKRQAKHKQ**RKR**L**K**S to** KR**EKRQAKHKQ**AAA**L**K**S) in exon 3 is denoted by AAA. (b) After a *Drd*I digest and Southern hybridization using a 3′ flanking probe complementary to the region indicated with the striped box, the wild type band was 13.8 kb and the targeted allele band was 6.1 kb. (c) To verify male germline self-excision of the *neo* cassette, PCR was performed using primers that bound to *BMP2* outside the *neo* cassette. PCR produced a 525 bp band from wild type DNA and a 607 bp if the targeting vector was inserted and the *neo* cassette excised, leaving a LoxP sequence. (d) Expression levels of conventional BMP2 were measured in skeletal muscle by western blot of cytoplasmic extracts using an anti-BMP2 antibody. The bands representing BMP2 proprotein, precursor of secreted BMP2, were quantified digitally and mutants were normalized to wild types within each experiment before averaging between experiments.  *n* = 4  for wild type gastrocnemius,  *n* = 5  for mutant gastrocnemius, and  *n* = 3  for both wild type and mutant quadriceps.

Having demonstrated in cell culture that the **RKR** to **AAA** mutation blocked nuclear localization of nBMP2 while still allowing secretion of normal quantities of the conventionally processed and secreted BMP2 growth factor, we constructed a targeting vector and generated  nBmp2NLS^tm^  mutant mice bearing the **RKR** to **AAA** mutation (Figure 2). Both heterozygous and homozygous mutant mice appeared morphologically normal and were fertile.

### 3.2. Verification of Normal Secreted BMP2 Function in nBmp2NLS^tm^ Mice

Work by others predicted that the **RKR** to **AAA** mutation would produce secreted BMP2 that was still able to bind its receptors, activate downstream genes, and induce ectopic bone formation, but that it might have increased diffusion range through the extracellular matrix [31, 32]. Increased diffusion range could disrupt embryonic patterning, but the normal appearance of the mutant mice ruled out major patterning abnormalities. Any minor patterning abnormalities would likely be manifest in the skeleton, since secreted BMP2 plays an important role in skeletal development and limb patterning. Skeletal preparations of wild type and homozygous mutant mice were performed and digital caliper measurements were taken of limbs, pelvis, scapula, ribcage, skull, and vertebrae, with multiple measurements at various positions on each bone. No significant differences were detected between wild type and mutant mice in any of the bones measured, supporting the conclusion that the function of secreted BMP2 is not impaired in  nBmp2NLS^tm^  mice (see Supplemental Table  1 in Supplementtary Materials available online at http://dx.doi.org/10.1155/2013/125492).

### 3.3. Upside-Down Hanging Ability Is Decreased in nBmp2NLS^tm^ Mice

**Figure 3:**
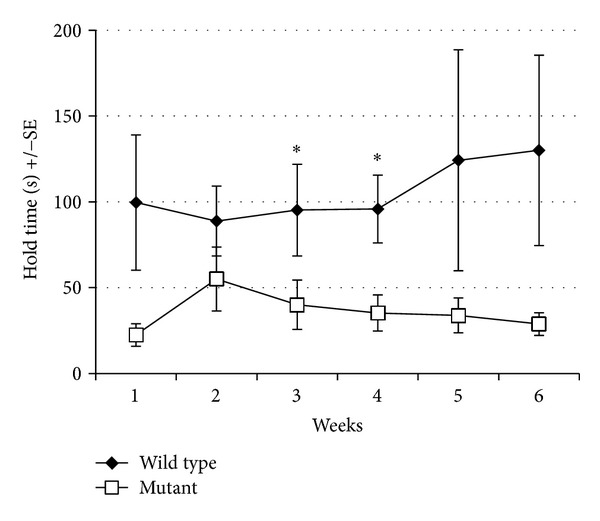
The  nBmp2NLS^tm^  mice show impaired performance in the grip/strength test. The length of time mice could hold to the underside of a wire mesh was measured once a week for six weeks in wild type and  nBmp2NLS^tm^  mice. Data are presented as mean ± SEM and *denotes  *P* < 0.05;  *n* = 5  per group.

Because  nBmp2NLS^tm^  mice appeared phenotypically normal, a series of preliminary tests were performed to detect subtle phenotypic changes. In one evaluation, five wild type and five mutant mice were tested once a week for six weeks to measure the length of time they could cling to the underside of a wire mesh cage lid [26]. On average, wild type mice held on 2-3 times as long as mutants did at every time point, and the difference was significant (*P* < 0.05) at weeks 3 and 4 (Figure 3). This trend suggested that we should consider the possibility of a neuromuscular defect in  nBmp2NLS^tm^  mice [26].

### 3.4. nBMP2 Is Detectable in Myonuclei of Wild Type but Not nBmp2NLS^tm^ Mice

**Figure 4:**
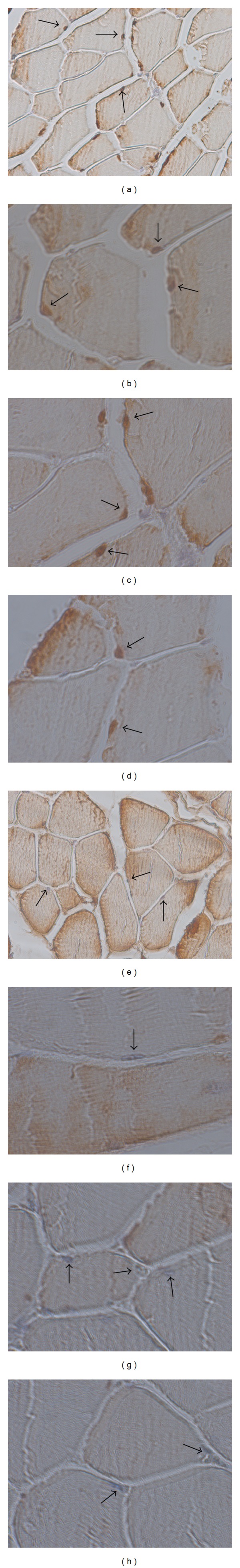
nBMP2 is detectable in myonuclei of wild type but not  nBmp2NLS^tm^  mice. Gastrocnemius muscles were sectioned and stained by immunohistochemistry. (a)–(d) are wild type; (e)–(h) are mutant. (a) and (e) show sections at 40x magnification, and all other panels show 100x. The brown BMP2 antibody stain is evident in myonuclei of wild type muscle, whereas myonuclei stain pale blue in the mutant muscle. Arrows indicate some (but not all) of the myonuclei.

To examine the expression of nBMP2 and BMP2 in skeletal muscle, gastrocnemius muscle from wild type and mutant mice was paraffin embedded, cross-sectioned, and stained using a biotin-tagged primary antibody against BMP2 (NBP1-19751G from Novus) and a Dako visualization system. The myotubes in skeletal muscle are long, multinucleated cells with the myonuclei positioned against the outer edges of the myotube. Brown BMP2 staining of myonuclei was detectable in wild type but not  nBmp2NLS^tm^  muscle, whereas nonnuclear BMP2 staining was detectable in both wild type and mutant. This result validated the effectiveness of the targeted NLS-inactivating mutation in a whole animal, and it also demonstrated that nBMP2 is expressed in skeletal muscle of control animals (Figure 4).

The presence of extranuclear BMP2 staining in both wild type and mutant skeletal muscle suggested that the expression of conventional BMP2 was not inhibited by the  nBmp2NLS^tm^  mutation. To further examine expression levels of the conventional protein, cytoplasmic extracts were prepared from gastrocnemius and quadriceps muscles and examined by western blot. The BMP2 proprotein, precursor of secreted BMP2, is detectable as a 50 kDa band. Levels of the proprotein were not different between wild type and mutant, again suggesting unaltered expression of conventional BMP2 (Figure 2(d)).

### 3.5. Skeletal Muscle Relaxation Times Are Prolonged in nBmp2NLS^tm^ Mice

**Figure 5:**
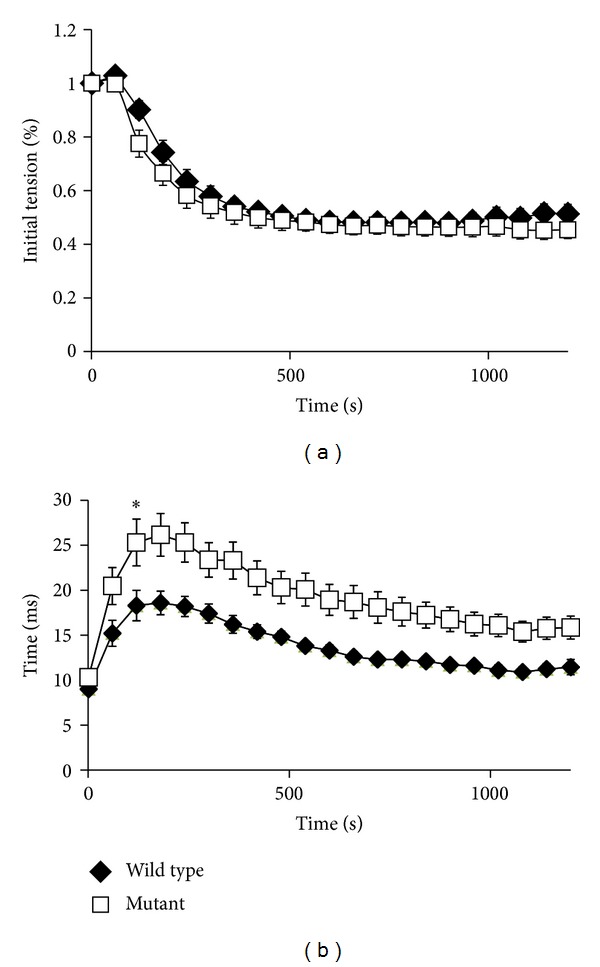
Muscle function is altered in  nBmp2NLS^tm^  mice. (a) Muscle fatigue in both control and  nBmp2NLS^tm^  GPS muscle complex was similar in response to 2 Hz twitch contractions for 10 minutes. (b) Muscle relaxation was significantly delayed in nBmp2NLS^tm^ muscle compared to WT control muscle beginning at 60 seconds and persisting throughout the 10 minutes in which these measurements were made. Data are presented as mean ± SEM and * denotes  *P* < 0.05,  *n* = 7  per group. Only the first significant difference is marked, but all values from 60 seconds on were significantly different from control.

To examine the possibility of a neuromuscular defect, we measured muscle performance *in situ*. The peak tetanic tension of the gastrocnemius, plantaris, and soleus (GPS) muscle group was slightly but significantly higher in  nBmp2NLS^tm^  mice compared to control mice when normalized to muscle weight (Table 1). No significant differences were observed in the peak twitch tension after normalization to muscle weight (Table 1), and no significant differences were observed in muscle fatigability in response to a 10 minute, 2 Hz twitch protocol (Figure 5(a)).

**Figure 6:**
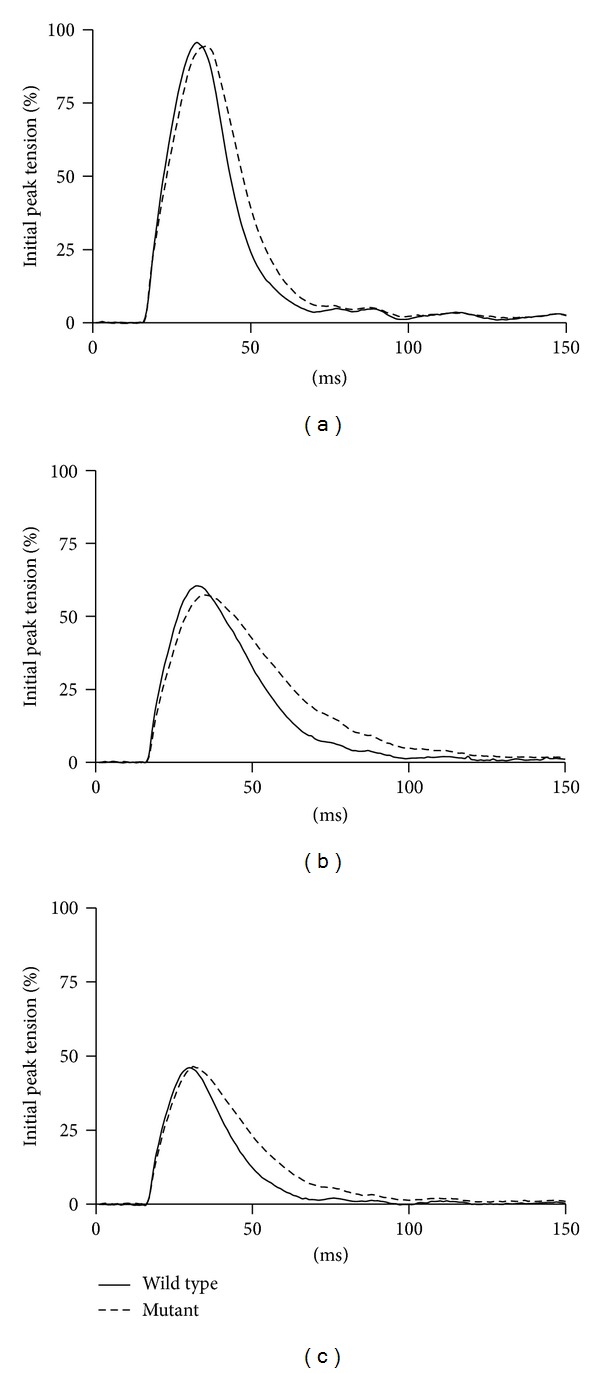
Slowed muscle relaxation is evident in twitch contractions. The average muscle twitch contraction for both wild type and  nBmp2NLS^tm^  mice is shown for the initial contractions (a), the contraction following 2 minutes of stimulation (b), and the contraction following 6 minutes of stimulation (c). Each trace represents the average of 7 contractions.

A significant difference did emerge, however, when relaxation kinetics were examined. Half-relaxation times were prolonged by up to 42% in the  nBmp2NLS^tm^  muscle compared to control (Figure 5(b)). This prolonged relaxation time was apparent in averaged twitch performance traces from initial (Figure 6(a)), 2 min (Figure 6(b)), and 6 min (Figure 6(c)) time points in the 2 Hz twitch contraction protocol.

### 3.6. Force Frequency Analysis Exhibits a Shift in nBmp2NLS^tm^ Mouse Skeletal Muscle

When muscle twitches are stimulated at increasing frequencies *in situ*, the contractions eventually become so frequent that the muscle is unable to complete one relaxation before the next contraction begins, and twitch peak traces begin to merge. The force or tension generated by the muscle increases as twitch peaks merge, until force reaches its maximum and thereafter remains level regardless of additional increases in the stimulation frequency. This could be considered the *in situ* version of a muscle cramp. Slowed relaxation after contraction widens each twitch peak, causing peaks to merge at a lower stimulation frequency, shifting the force frequency curve leftward.

**Figure 7:**
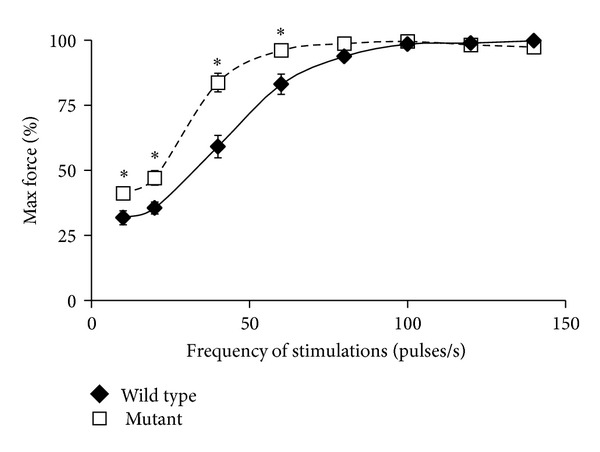
Force frequency analysis of control and  nBmp2NLS^tm^  muscle. A significant shift in the force frequency curve was observed in  nBmp2NLS^tm^  muscle compared to wild type, supporting the evidence for slowed relaxation in mutants. Data are presented as mean ± SEM and *denotes  *P* < 0.01,  *n* = 6  per group.

Because the  nBmp2NLS^tm^  mice showed slowed relaxation after contraction, we performed a force frequency analysis. The  nBmp2NLS^tm^  mutant mice generated significantly elevated relative forces at 10, 20, 40, and 60 Hz (*P* < 0.01). The data collected fit an expected sigmoidal curve and showed a significant leftward shift as determined by the average force achieved at each frequency compared to the control (Figure 7). This shift in the force frequency pattern, as well as the slowed relaxation rates after twitch contractions, suggests impairment of Ca^2+^ sequestration by the sarcoplasmic reticulum (SR) after muscle contraction.

### 3.7. SERCA Activity Is Reduced in nBmp2NLS^tm^ Skeletal Muscle

**Figure 8:**
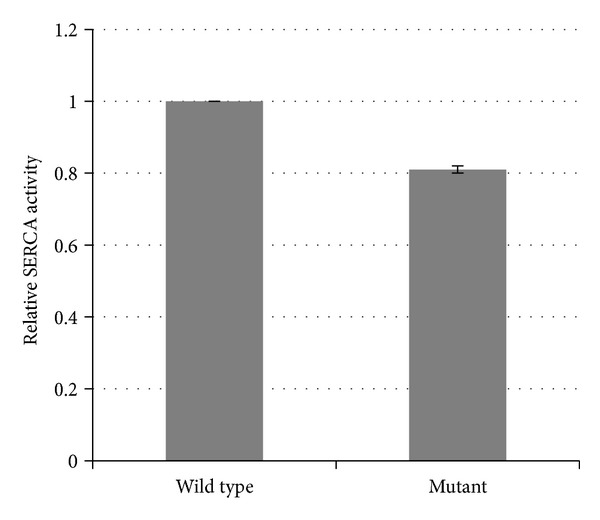
SERCA activity is reduced in  nBmp2NLS^tm^  muscle compared to controls. SR was isolated from control and mutant muscle and Ca^2+^ ATPase activity was measured. Data are presented as mean ± SEM,  *P* < 0.05,  *n* = 3  per group.

The relaxation phase of a skeletal muscle contraction occurs when Ca^2+^ is pumped in an ATP-dependent manner from the cytosol back into the SR by sarco/endoplasmic reticulum Ca^2+^ ATPases (SERCA). Measurement of SERCA activity in skeletal muscle revealed that SERCA activity in  nBmp2NLS^tm^  mice is reduced to 81 ± 1% of wild type activity (Figure 8). This observation is consistent with the slowed relaxation rate observed in  nBmp2NLS^tm^  muscle.

## 4. Discussion

Several members of the BMP protein family are expressed in variant forms that are translocated to the nucleus rather than being secreted from the cell [20]. A major challenge in characterizing the function of these nuclear variants of BMPs is that the nuclear protein is translated from the same mRNA transcript as the conventional secreted BMP2 growth factor, as demonstrated by our prior observation that both nBMP2 and BMP2 are produced from a transfected wild type BMP2 cDNA expression vector in tissue culture [20]. This precludes usage of several of the most common methods for studying gene function, including RNAi, quantitative RT-PCR, and gene knockout, which would all affect and/or detect the conventional secreted BMP2 growth factor as well as nBMP2. Instead, we knocked in an **RKR** to **AAA** mutation in the bipartite NLS to prevent nuclear localization of nBMP2 while allowing production of secreted BMP2.

The **RKR** to **AAA** mutation has minimal effect on the function of secreted BMPs. BMP2 growth factor that was truncated N-terminally of either the R, K, or R still had the capability to induce ectopic bone formation to the same extent as wild type BMP2 [31]. Moreover, deletion or mutation of the homologous sequence in BMP4 produced a protein that was functionally indistinguishable from wild type BMP4 in receptor binding, antagonist binding, and target gene responsiveness [32]. Animal cap conjugation experiments with Xenopus embryos, however, suggested that these mutations reduced the ability of Bmp4 to bind heparin sulfate in the extracellular matrix, with the result of increased diffusion range [32]. Together, these experiments predicted that BMP2 bearing the same mutation (**RKR** to **AAA**) would also function normally in terms of receptor binding and target gene activation but might have an increased diffusion range.

Secreted BMP2 plays a critical role in dorsal-ventral patterning of the early embryo, in limb patterning, and in skeletogenesis, so an increased diffusion range of the secreted growth factor is likely to cause morphological abnormalities in mice [33–35]. The  nBmp2NLS^tm^  mouse, however, displayed no morphological or patterning abnormalities, and no differences in the length, width, thickness, or shape of any of the bones measured, indicating that there is no physiologically significant alteration in the diffusion range of BMP2 in these mice. We conclude that the **RKR** to **AAA** mutation in the  nBmp2NLS^tm^  mouse preserved the function of the secreted BMP2 growth factor. The  nBmp2NLS^tm^  mouse, therefore, is a good model for studying the molecular and physiological functions of nBMP2 as distinct from secreted BMP2.

The  nBmp2NLS^tm^  mouse showed decreased ability to cling upside-down to a wire mesh cage lid, and immunohistochemistry showed that nBMP2 is detectable in skeletal muscle myonuclei of wild type but not mutant animals. Mutant muscle showed no increase in fatigability *in situ*, however, suggesting that the reduced hang time is not related to simple muscle fatigue. Instead, the shifted force frequency curve and slowed relaxation rates in the mutant muscle suggest that mutant mice may be more prone to muscle cramping if contractions merge during intense muscle activity as they do during high-frequency *in situ* stimulations. Slowed muscle relaxation does lead to cramping in the human syndrome Brody myopathy, where the length of time required for pumping Ca^2+^ back into the SR after each contraction is increased due to mutations in the gene for SERCA1, the pump that performs this function in skeletal muscle [36–40]. Brody patients have also reported problems with their hands “locking” after making a tight fist [40]. This cramping can be painful, is most frequent after exercise, and grows worse with age. The slowed relaxation rates in both Brody patients and  nBmp2NLS^tm^  mice suggest the possibility that nBMP2 may function in some aspect of the SERCA1 production/activation pathway.

Our demonstration of reduced SERCA1 activity in the  nBmp2NLS^tm^  mouse supports this hypothesis. The phenotype of the  nBmp2NLS^tm^  mouse, however, could also result from the dysfunction of several other proteins, because muscle contraction/relaxation cycles are quite complex. Contractions occur when an action potential leads to activation of Ca^2+^ release channels (ryanodine receptors) in the SR membrane. Ca^2+^ floods into the cytoplasm and binds troponin C, which modulates the function of tropomyosin so that myosin can bind strongly to actin and cause shortening of the sarcomere. The contraction ceases when active pumping (by SERCA1) of Ca^2+^ back into the SR depletes cytoplasmic Ca^2+^ levels, causing release of Ca^2+^ from troponin C [41, 42]. The  nBmp2NLS^tm^  phenotype could be caused by leaky ryanodine receptors (RyR) in the SR membrane, which would lead to prolonged elevation of Ca^2+^ in the cytosol even if the SERCA pumps were working normally. An increased affinity of troponin C for Ca^2+^ could also cause slowed muscle relaxation by increasing the level of cytosolic Ca^2+^ depletion that would have to occur before Ca^2+^ was released from troponin C. Ongoing work in our lab on other physiological pathways that are controlled by intracellular Ca^2+^ movement, including neuronal signaling, immune system activation, and cell cycle regulation, indicates that these pathways are also disrupted in  nBmp2NLS^tm^  mice. We predict, therefore, that nBMP2 affects aspects of intracellular Ca^2+^ transport that are shared among multiple pathways rather than those (such as troponin C) that are unique to skeletal muscle.

## 5. Conclusions

In summary, the muscle phenotype that we identified in the  nBmp2NLS^tm^  mouse suggests that a significant alteration in intracellular Ca^2+^ transport resulted from impeded nuclear localization of nBMP2. The prolonged relaxation kinetics, the shift in the force frequency curve, and the reduction in SERCA activity are most consistent with a condition of delayed Ca^2+^ uptake by the SR, although other possibilities have not been ruled out. We have recently observed (unpublished data) that  nBmp2NLS^tm^  mice also have cognitive deficits that are consistent with an intracellular Ca^2+^-handling defect in hippocampus. It is likely that Ca^2+^ handling is disrupted in other tissues as well and that nBMP2 will be found to function in multiple molecular pathways that govern intracellular Ca^2+^ transport. The  nBmp2NLS^tm^  mouse constitutes a valuable model for characterizing the functions of nBMP2 relative to Ca^2+^ transport pathways and for distinguishing those functions from the activities of secreted BMP2.

## Supplementary Material

nBmp2NLS^tm^ mice show normal skeletal structure. In order to check for subtle differences between skeletal structure in wild type and nBmp2NLS^tm mice, digital calipers were used to measure bone dimensions on skeletal preparations of wild type and mutant male mice. Measurements were taken at various points on the femur, tibia, humerus, radius, spinal column, pelvis, rib cage, and skull. The P value for wild type compared to mutant was greater than 0.05 for every measurement.

## Figures and Tables

**Table 1:** Body weight, age, and muscle force characteristics.

	Control (*n* = 7)	nBmp2NLS^tm^ (*n* = 7)
Age (weeks)	50.9 ± 0.4	43.9 ± 7.5
Body weight (g)	36.9 ± 1.1	35.3 ± 2.4
GPS muscle weight (mg)	160.9 ± 9.6	171.7 ± 4.3
Peak tetanic tension (g)	498 ± 30	567 ± 18
Peak tetanic tension (g/mg muscle)	3.09 ± 0.05	3.31 ± 0.08*
Peak twitch tension (g)	192.6 ± 9.7	220.6 ± 5.1*
Peak twitch tension (g/mg muscle)	1.20 ± 0.03	1.29 ± 0.04

Data are presented as means ± SEM. Where indicated, *indicates *P* < 0.05 compared to control values.

## References

[1] Urist MR (1965). Bone: formation by autoinduction. *Science*.

[2] Wozney JM, Rosen V, Celeste AJ (1988). Novel regulators of bone formation: molecular clones and activities. *Science*.

[3] Chen D, Zhao M, Mundy GR (2004). Bone morphogenetic proteins. *Growth Factors*.

[4] Goldstein AM, Brewer KC, Doyle AM, Nagy N, Roberts DJ (2005). BMP signaling is necessary for neural crest cell migration and ganglion formation in the enteric nervous system. *Mechanisms of Development*.

[5] Hogan BL (1996). Bone morphogenetic proteins: multifunctional regulators of vertebrate development. *Genes and Development*.

[6] Mishina Y (2003). Function of bone morphogenetic protein signaling during mouse development. *Frontiers in Bioscience*.

[7] Schlange T, Arnold H-H, Brand T (2002). BMP2 is a positive regulator of Nodal signaling during left-right axis formation in the chicken embryo. *Development*.

[8] Zhang H, Bradley A (1996). Mice deficient for BMP2 are nonviable and have defects in amnion/chorion and cardiac development. *Development*.

[9] Kawamura C, Kizaki M, Ikeda Y (2002). Bone morphogenetic protein (BMP)-2 induces apoptosis in human myeloma cells. *Leukemia and Lymphoma*.

[10] Raida M, Clement JH, Ameri K, Han C, Leek RD, Harris AL (2005). Expression of bone morphogenetic protein 2 in breast cancer cells inhibits hypoxic cell death. *International Journal of Oncology*.

[11] Du Y, Yip H (2010). Effects of bone morphogenetic protein 2 on Id expression and neuroblastoma cell differentiation. *Differentiation*.

[12] Guimond J-C, Lévesque M, Michaud P-L (2010). BMP-2 functions independently of SHH signaling and triggers cell condensation and apoptosis in regenerating axolotl limbs. *BMC Developmental Biology*.

[13] Rider CC, Mulloy B (2010). Bone morphogenetic protein and growth differentiation factor cytokine families and their protein antagonists. *Biochemical Journal*.

[14] Constam DB, Robertson EJ (1999). Regulation of bone morphogenetic protein activity by pro domains and proprotein convertases. *Journal of Cell Biology*.

[15] Wozney JM (1989). Bone morphogenetic proteins. *Progress in Growth Factor Research*.

[16] Hiyama A, Gogate SS, Gajghate S, Mochida J, Shapiro IM, Risbud MV (2010). BMP-2 and TGF-*β* stimulate expression of *β*1,3-glucuronosyl transferase 1 (GlcAT-1) in nucleus pulposus cells through AP1, TonEBP, and Sp1: role of MAPKs. *Journal of Bone and Mineral Research*.

[17] Alarcón C, Zaromytidou A-I, Xi Q (2009). Nuclear CDKs drive Smad transcriptional activation and turnover in BMP and TGF-*β* pathways. *Cell*.

[18] Lemonnier J, Ghayor C, Guicheux J, Caverzasio J (2004). Protein kinase C-independent activation of protein kinase D is involved in BMP-2-induced activation of stress mitogen-activated protein kinases JNK and p38 and osteoblastic cell differentiation. *Journal of Biological Chemistry*.

[19] Moustakas A, Heldin C-H (2002). From mono- to oligo-Smads: the heart of the matter in TGF-*β* signal transduction. *Genes and Development*.

[20] Felin JE, Mayo JL, Loos TJ (2010). Nuclear variants of bone morphogenetic proteins. *BMC Cell Biology*.

[21] Copeland NG, Jenkins NA, Court DL (2001). Recombineering: a powerful new tool for mouse functional genomics. *Nature Reviews Genetics*.

[22] Warming S, Costantino N, Court DL, Jenkins NA, Copeland NG (2005). Simple and highly efficient BAC recombineering using galK selection. *Nucleic Acids Research*.

[23] Bunting M, Bernstein KE, Greer JM, Capecchi MR, Thomas KR (1999). Targeting genes for self-excision in the germ line. *Genes and Development*.

[24] George SH, Gertsenstein M, Vintersten K (2007). Developmental and adult phenotyping directly from mutant embryonic stem cells. *Proceedings of the National Academy of Sciences of the United States of America*.

[25] Mansour SL, Thomas KR, Capecchi MR (1988). Disruption of the proto-oncogene int-2 in mouse embryo-derived stem cells: a general strategy for targeting mutations to non-selectable genes. *Nature*.

[26] Papaioannou VE, Behringer RR (2005). *Mouse Phenotypes—A Handbook of Mutation Analysis*.

[27] Hancock CR, Brault JJ, Wiseman RW, Terjung RL, Meyer RA (2005). 31P-NMR observation of free ADP during fatiguing, repetitive contractions of murine skeletal muscle lacking AK1. *The American Journal of Physiology*.

[28] Hancock CR, Janssen E, Terjung RL (2005). Skeletal muscle contractile performance and ADP accumulation in adenylate kinase-deficient mice. *The American Journal of Physiology*.

[29] Hancock CR, Janssen E, Terjung RL (2006). Contraction-mediated phosphorylation of AMPK is lower in skeletal muscle of adenylate kinase-deficient mice. *Journal of Applied Physiology*.

[30] Kosk-Kosicka D, Lambert DG (1999). Calcium signaling protocols. *Methods in Molecular Biology*.

[31] Hillger F, Herr G, Rudolph R, Schwarz E (2005). Biophysical comparison of BMP-2, ProBMP-2, and the free pro-peptide reveals stabilization of the pro-peptide by the mature growth factor. *Journal of Biological Chemistry*.

[32] Ohkawara B, Iemura S, ten Dijke P, Ueno N (2002). Action range of BMP is defined by its N-terminal basic amino acid core. *Current Biology*.

[33] de Robertis EM (2009). Spemann’s organizer and the self-regulation of embryonic fields. *Mechanisms of Development*.

[34] Maatouk DM, Choi K-S, Bouldin CM, Harfe BD (2009). In the limb AER Bmp2 and Bmp4 are required for dorsal-ventral patterning and interdigital cell death but not limb outgrowth. *Developmental Biology*.

[35] Robert B (2007). Bone morphogenetic protein signaling in limb outgrowth and patterning. *Development Growth and Differentiation*.

[36] Odermatt A, Barton K, Khanna VK (2000). The mutation of Pro789 to Leu reduces the activity of the fast-twitch skeletal muscle sarco(endo)plasmic reticulum Ca^2+^ ATPase (SERCA1) and is associated with Brody disease. *Human Genetics*.

[37] Odermatt A, Taschner PE, Khanna VK (1996). Mutations in the gene-encoding SERCA1, the fast-twitch skeletal muscle sarcoplasmic reticulum Ca^2+^ ATPase, are associated with Brody disease. *Nature Genetics*.

[38] Benders AA, Veerkamp JH, Oosterhof A (1994). Ca^2+^ homeostasis in Brody’s disease. A study in skeletal muscle and cultured muscle cells and the effects of dantrolene and verapamil. *Journal of Clinical Investigation*.

[39] Brody IA (1969). Muscle contracture induced by exercise. A syndrome attributable to decreased relaxing factor. *The New England Journal of Medicine*.

[40] Zhang Y, Fujii J, Phillips MS (1995). Characterization of cDNA and genomic DNA encoding SERCA1, the Ca^2+^-ATPase of human fast-twitch skeletal muscle sarcoplasmic reticulum, and its elimination as a candidate gene for Brody disease. *Genomics*.

[41] Endo M (2009). Calcium-induced calcium release in skeletal muscle. *Physiological Reviews*.

[42] Betzenhauser MJ, Marks AR (2010). Ryanodine receptor channelopathies. *Pflugers Archiv*.

